# The load-velocity profiles and exercise-specific velocity zones for seven commonly used weightlifting exercises

**DOI:** 10.1371/journal.pone.0352209

**Published:** 2026-07-06

**Authors:** Jonathon Weakley, Tandia Wood, Amador García-Ramos, Tayah R. Brennan, Chieh-Ying Chiang, Liam Schultz, J. Bryan Mann, Matthew Morrison, Daniela Gonçalves Pedrosa, Mark W. Creaby

**Affiliations:** 1 School of Behavioural and Health Sciences, Australian Catholic University, Brisbane, Queensland, Australia; 2 Sports Performance, Recovery, Injury and New Technologies (SPRINT) Research Centre, Australian Catholic University, Brisbane, Queensland, Australia; 3 Carnegie Applied Rugby Research (CARR) Centre, Carnegie School of Sport, Leeds Beckett University, Leeds, United Kingdom; 4 Department of Physical Education and Sport, University of Granada, Granada, Spain; 5 Department of Sports Sciences and Physical Conditioning, Universidad Catolica de la Santisima Concepcion, Concepcion, Chile; 6 Department of Sports Science Training - Combat, National Taiwan Sport University, Taiwan; 7 Taiwan Institute of Sports Science, Kaohsiung City, Taiwan; 8 Department of Kinesiology and Sport Management, Texas A&M University, College Station, Texas, United States of America; 9 Centre for Human Factors and Systems Science, University of the Sunshine Coast, Sippy Downs, Queensland, Australia; 10 Independent Researcher; University of Vic - Central University of Catalonia: Universitat de Vic - Universitat Central de Catalunya, SPAIN

## Abstract

Velocity zones (e.g., 1.0–0.75 m·s^-1^) are commonly aligned with terminology such as “starting strength”, ‘speed-strength’, ‘strength-speed’, ‘accelerative strength’, or ‘absolute strength’. However, the load-velocity profiles of most exercises do not align with these discrete bands. The aims of this study were to 1) develop load-velocity profiles of seven weightlifting derivatives; and 2) create exercise-specific velocity zones that can be used to guide training prescription. Fourteen (6 males and 8 females) weightlifting athletes undertook six testing sessions that required maximal strength testing on occasions one and two, and the development of load-velocity profiles for the power snatch, hang power clean, snatch pull, hang clean pull, hang power snatch, clean pull, and hang snatch pull on testing occasions three to six. During each testing occasion, peak velocity was assessed. Linear mixed models with effect size ±95% confidence limits (CL) were used to detect changes across profiles and estimate exercise specific velocity zones. While all load-velocity profiles had a clear reduction in velocity as load was increased, each exercise was found to have substantially different velocity zones when compared to previous recommendations. Of note, all ‘absolute strength’ zones (i.e., > 80% one repetition maximum) from the weightlifting derivatives were found to be greater than 1.3 m·s^-1^ which is commonly used as the threshold for ‘starting strength’. These findings demonstrate that, if these terms are to be used, exercise-specific load-velocity profiles should be developed. Furthermore, these findings provide practitioners with exercise-specific zones that can be used to enhance training prescription and target specific strength qualities.

## Introduction

Resistance training is a commonly implemented training method that supports muscle strength, power, and hypertrophy [1,2]. Traditionally, it is prescribed through athletes completing a maximal strength test (e.g., a one repetition maximum (1RM)) and a percentage of this maximal load being prescribed for a fixed number of repetitions (e.g., 10 repetitions at 70% of 1RM). However, due to the near perfect inverse relationship between load and velocity, exercise velocity can also be used to support training prescription [3–5]. The use of velocity during resistance training is commonly implemented through load-velocity profiling of athletes and can be done across a wide range of exercises, including compound non-ballistic (e.g., back squat) and weightlifting and derivative movements (e.g., power snatch, clean pull) [3,4,6]. These load-velocity profiles are intended to support training prescription and direct measurement of maximal strength qualities [7,8]. Although some practitioners use load–velocity relationships to estimate 1RM values, this approach can be limited and may not provide sufficient accuracy [9]. For this reason, practitioners can focus on velocity-zones during prescription. However, there are large differences in the velocities exhibited during training and performance depending upon the exercise being completed. This can be attributed to differences in relative strength, the task being completed, or the technical proficiency.

Terms such as “starting strength”, ‘speed-strength’, ‘strength-speed’, ‘accelerative strength’, and ‘absolute strength’ are commonly used by practitioners and throughout the scientific literature to refer to different strength qualities [10–12]. These qualities are said to be trained when exercising at different mean concentric velocities (e.g., > 1.3 m·s^-1^; 1.0–1.3 m·s^-1^; 1.0–0.75 m·s^-1^; 0.75–0.5 m·s^-1^; and <0.5 m·s^-1^, respectively) and intensities [12–14]. However, these generic terms and velocity ranges have very little value, except to a small subset of exercises which move within these velocity zones (e.g., the back squat) [15]. This is an issue for weightlifting and associated derivative exercises, which are monitored through peak concentric velocity rather than mean concentric velocity [16,17], has the barbell moving independently from the athlete [18], and rarely have repetitions that go below a mean concentric velocity of 1.3 m·s^-1^ [6]. Thus, by using these zones and terminologies, athletes using weightlifting related movements would nearly always be training “starting strength”. These movements are commonly used by athletes and having clear terminology may support training prescription and monitoring [18–21]. It should be noted that previous research examining weightlifting derivatives has reported barbell velocities across a range of loads (e.g., Roman [14] and Suchomel et al., [18]), demonstrating that these exercises exhibit substantially higher peak velocities than traditional resistance-training movements. However, these studies did not establish exercise‑specific velocity zones or characterize these zones across multiple derivatives within the same cohort. Consequently, although prior work provides valuable foundational information, further profiling is required to develop clear, exercise‑specific velocity bands that can support applied prescription. Therefore, there is a need to profile the load-velocity relationship of these different weightlifting movements and provide information regarding what velocities these different strength qualities occur at.

Lifting velocity is commonly used to guide and monitor resistance training prescription and performance [16,22,23]. Furthermore, terms such as ‘speed-strength’ and ‘accelerative strength’ are regularly used to describe training at a given relative intensity and are often aligned with generic velocity bands (e.g., ‘speed-strength occurs at 1.0-1.3 m·s^-1^’) [17]. However, each exercise has their own range of velocities that an athlete can exercise at, and the velocity zones commonly prescribed only align with a small subset of exercises [15]. This is particularly pertinent for weightlifting derivatives which are executed at high velocities (e.g., > 2.0 m·s^-1^) [6]. Therefore, using weightlifting athletes, the aims of this study were to 1) develop load-velocity profiles of seven weightlifting derivatives; and 2) create exercise specific velocity zones that can be used to guide training prescription. It was hypothesized that there will be reductions in velocity as load is increased. However, there will be differences in the velocities observed and those previously recommended for the specific targeting of strength qualities.

## Methods and materials

### Experimental approach to the problem

A repeated-measures, within-participants design was used to develop load-velocity profiles and exercise-specific velocity zones for seven weightlifting derivatives between 6^th^ of January and 12^th^ of December 2024. Fourteen weightlifting athletes completed six testing sessions. During sessions one and two, athletes underwent one-repetition maximum (1RM) assessments for the power snatch, snatch pull, hang power snatch, hang snatch pull, hang power clean, clean pull, and hang clean pull. In sessions three through six, athletes performed each lift with loads ranging from 20% to 100% of their individual 1RM. Peak concentric barbell velocity was measured using a Perch device, and load-velocity profiles were constructed from these data.

### Participants

Fourteen participants (six males and eight females; males age: 29.5 ± 2.6 years, height: 1.72 ± 0.08 m, body mass: 82.6 ± 5.7 kg; females age: 28.4 ± 4.4 years, height: 168.6 ± 5.6 cm, body mass: 69.7 ± 5.3 kg) volunteered to participate in the study. To estimate the minimum number of participants required to detect within‑subject changes in peak velocity across a load–velocity profile, an a priori power calculation was performed based on a paired (within-subject) comparison between relative loads, assuming a moderate effect size (d = 0.5), a statistical power of 0.80, and an alpha level of 0.05 [24]. This analysis indicated that a minimum of 11 participants would be required and was conducted using the *pwr* package in R (version 4.4.2). It is acknowledged that this power calculation does not explicitly account for the full linear mixed-effects modelling framework, sex-specific effects, or within-subject correlation structures; however, it was used as a conservative estimate to support sample adequacy. The repeated-measures nature of the data was fully addressed in the primary analyses through the inclusion of participant-specific random intercepts in the linear mixed-effects models.

To be included in the study, all participants were required to be over 18 years of age, injury free, have a relative back squat strength >1.5 body mass, have at least two years of weightlifting experience, and actively competing in weightlifting competitions. Participants were excluded if they had sustained any injuries within the last six months. All participants were provided with the opportunity to ask questions and provided written informed consent prior to the start of the first testing occasion. This project was approved by the Australian Catholic University Human Research Ethics Committee (Ethics number: 2019-131H).

### Procedures

All testing was completed in the same biomechanics laboratory for the course of the project. Participants attended six sessions, with a minimum rest of 24 hours and a median of five days between testing occasions. Upon arrival to the first testing session, athletes were provided information relating to the study and provided informed consent. Height and body mass were then recorded using a Seca 217 portable stadiometer (SECA, Hamburg, Germany) and Seca 803 Weight Scales (SECA, Hamburg, Germany), respectively. For all testing sessions, participants completed their normal warm up, which included foam rolling, mobility, banded activations, and movement with an empty barbell. Warm up times were approximately 20–30 minutes. During all testing sessions, participants were asked to complete repetitions with maximal intent, followed by two minutes rest between sets. In sessions one and two, all participants completed 1RM testing of the seven exercises that involved increasing load on the barbell until a maximal load that they could lift was found. The progressive loading process involved the athlete and research team making informed load selections based off recent training history and live velocity data [25]. If a repetition was unsuccessful, the last successful attempt was used as their 1RM for the given movement. On testing occasion one, the power snatch, hang power clean, snatch pull, and hang clean pull were assessed. On testing occasion two, the hang power snatch, clean pull, and hang snatch pull were tested. For feasibility, several strength tests were completed on each day, as previous research has demonstrated this does not influence maximal testing validity [26]. After each exercise was tested, a rest period of ~10 minutes was provided followed by the next exercise being progressively loaded. 1RMs were typically found after 3–5 attempts and it should be noted that all participants completed exercises in the same order to ensure consistency across participants.

During sessions three to six, participants completed three repetitions at 20%, 40% and 60%, and one repetition at 80%, and 90% of the established 1RM. In two of these sessions the power snatch, hang power clean, snatch pull, and hang clean pull were assessed; the hang power snatch, clean pull, and hang snatch pull were assessed in the other two sessions. These exercises were selected as they are commonly used within the participants training programs. Furthermore, they were completed in the above order. It should be noted that these sessions and the order of the exercises in these sessions were standardized and consistent. Following their warm-up, the athletes progressively loaded the barbell, with each repetition requiring maximal intent.

### Completion of exercises

Details of the seven completed exercises are below. Participants used the same warm up prior to each session and footwear was consistent within and across sessions. Weightlifting straps were not permitted in the completion of the lifts. All sessions were completed within a biomechanics laboratory with repetitions visually recorded. It should be acknowledged that while some exercises (e.g., clean pull, snatch pull) are traditionally prescribed as a percentage of clean or snatch 1RM. Therefore, following the completion of all repetitions, visual inspection from a Level 2 Australian Strength and Conditioning Association (ASCA) accredited coach occurred to ensure that each repetition abided by the details provided below.

### Snatch pull and clean pull technique

The pull was defined as the movement of the barbell from the floor to the completion of full hip, knee, and ankle extension. Clean and snatch pulls were performed using similar movement characteristics, albeit differing in grip width.

For the snatch pull and clean pull, athletes used a stance approximately hip-width apart and the barbell over the foot. The bar was grasped with a pronated grip, arms positioned outside the thighs, and elbows rotated outward. The trunk was set in a neutral to slightly lordotic position, with hips slightly higher than the knees, shoulders over or marginally in front of the bar, and an initial trunk angle of approximately 35–45°. The first pull involved knee extension to move the bar from the floor to just below the knee, with the hips and shoulders rising together so that there was minimal change in trunk angle. Bar velocity increased progressively while the bar remained close to the body. As the bar passed the knee, a brief transition phase occurred, during which the knees extended to clear the bar followed by re-flexion.

The second pull was initiated from the thigh and characterized by rapid, coordinated extension of the hips, knees, and ankles. The bar was required to pass the thighs with little horizontal displacement, elbows remaining extended, and shoulder elevation occurring secondary to lower-body extension. At completion, full triple extension was required to be achieved with the trunk near vertical and heels elevated. The barbell was then released or decelerated in a controlled manner before subsequent repetitions. For repetitions to be deemed successful, a single clear movement was required.

### Hang power clean and hang power snatch technique

The hang power clean and hang power snatch began with the barbell initially positioned at the mid-thigh. For both exercises, participants adopted a stance between hip- and shoulder-width and maintained feet slightly externally rotated. A pronated grip was employed in both lifts. At the start position, elbows were fully extended and rotated slightly outward, heels remained flat, and the bar was held close to the body. The trunk was maintained rigid in a neutral to slightly lordotic posture, with shoulders over and slightly in front of the bar. The initial movement phase for both lifts consisted of a controlled eccentric lowering of the bar to just above the knees, immediately followed by a transition phase. During this transition, hips were displaced forward and the knees re-flexed slightly, repositioning the lower limbs under the bar and maintaining a close bar path and trunk rigidity.

The second pull for both movements was initiated at the mid-thigh, characterized by rapid and forceful extension of the hips, knees, and ankles, accompanied by elevation of the shoulders. The bar remained close to the thighs, with minimal horizontal displacement. In both lifts, elbow flexion occurred only after completion of triple extension: in the hang power clean, this facilitated pulling the body under the bar; in the hang power snatch, this facilitated movement under the bar as it was prepared to be received overhead.

In the hang power clean, the bar was received in a power (quarter-squat) position and racked across the anterior deltoids and clavicles, with elbows rotated forward and upward, forearms approximately parallel to the floor, and the torso upright. In contrast, during the hang power snatch, the bar was received overhead in a power position with elbows fully extended, arms aligned with the trunk and shins, and the head positioned slightly in front of the torso.

### Power snatch technique

The power snatch was performed from the floor with athletes using a stance between hip‑ and shoulder‑width with the feet slightly externally rotated and using a pronated grip wider than shoulder width, consistent with conventional snatch technique. At the start position, the barbell was positioned over the foot and kept close to the shins. The hips were set lower than the shoulders, the elbows fully extended and rotated slightly outward, and the heels flat. The trunk was rigid with a neutral to slightly lordotic spine. The head and neck were maintained in a neutral position, with the shoulders positioned over or marginally in front of the bar.

The first pull consisted of coordinated extension of the hips and knees to raise the bar from the floor to just below the knees while maintaining a near‑constant trunk angle. Throughout this phase, the bar was kept close to the shins and the shoulders and hips rose at a similar rate. As the bar passed the knees, athletes executed a brief transition phase characterised by forward displacement of the hips and slight re‑flexion of the knees.

The second pull was initiated as the bar reached the mid‑thigh. This phase was characterised by rapid, forceful extension of the hips, knees, and ankles, accompanied by vertical elevation of the shoulders. The bar remained close to the thighs with minimal horizontal displacement. Elbow flexion occurred only after completion of full triple extension, enabling the athlete to actively move under the rising bar.

The catch phase was performed in a power position. The bar was received overhead with the elbows fully extended, the arms aligned with the trunk and shins, and the head positioned slightly forward of the torso to support a stable overhead position. The torso remained upright with the feet flat and body mass centred over the mid‑foot.

### Hang snatch pull and hang clean pull technique

The hang snatch pull and hang clean pull were initiated from the mid‑thigh position, with athletes standing upright and the bar held close to the body. For both movements, athletes adopted a stance between hip‑ and shoulder‑width with the feet slightly externally rotated. A pronated snatch or clean grip was used, like those in the previously listed exercises. Elbows were extended and rotated slightly outward, the shoulders positioned over or slightly in front of the bar, and the trunk maintained in a neutral to slightly lordotic posture.

Participants were required to perform a controlled hip‑hinge to lower the bar to just above the patellae. During this eccentric phase, the hips moved posteriorly, the knees flexed slightly, and the bar remained close to the thighs. Then, without pausing, athletes transitioned back to the mid‑thigh position by re‑flexing the knees and displacing the hips forward, maintaining trunk rigidity and a consistent bar path.

The second pull commenced from the mid‑thigh and was characterized by rapid extension of the hips, knees, and ankles, accompanied by vertical shoulder elevation. In both lifts, the bar travelled close to the body with minimal horizontal displacement, and the elbows remained extended. Each repetition concluded upon full extension of the hips, knees, and ankles, with the repetition completed in a single clear motion. The bar was lowered under control to the mid‑thigh before the next repetition.

### Development of load-velocity profiles

The load-velocity profiles were constructed for each exercise consistent with the recommendations of Banyard et al., [27]. Additionally, velocity zones of starting strength, speed-strength, strength-speed, accelerative strength, and absolute strength were established at <25%, 25–45%, 45–65%, 65–80%, and >80% of 1RM load, respectively [11–14,17]. The reliability of peak concentric velocity at each intensity have been detailed by Wood et al., [28].

To develop the load-velocity profiles and establish relevant velocity zones for each exercise, a Perch device (Boston, MA, USA) was used. The Perch device is a commercially sold, camera-based system that has previously been validated [29]. The device was placed ~2.5 meters in front of the participant at a height of 2.1 meters on the crossbar of a weightlifting rack. The device measures barbell displacement at 30 Hz through a depth-perception camera that consists of three camera lenses within a single unit; while this sampling frequency may limit the temporal resolution of peak velocity detection, the device has demonstrated acceptable reliability and validity for assessing barbell velocity in resistance exercise [29]. The Perch was connected through Bluetooth to a Samsung Galaxy tablet (Samsung Electronics, Suigen, South Korea) that was running the Perch app (version 1.1.19) that recorded all values. At the completion of each session, all vertical peak velocity data were synced to the cloud, where the raw data files could be exported for further processing. All load-velocity profiles were developed with peak velocity, as this variable is commonly recommended for exercises that have ballistic intent (e.g., plyometric and weightlifting exercises) [16,17].

### Statistical analyses

All statistical analyses were conducted in R (version 4.4.2) and RStudio using the *tidyverse, lme4, MuMIn, emmeans,* and *ggplot2* packages. Data were grouped by exercise type, and linear mixed-effects models were fitted for each exercise using the *lmer* function from the *lme4* package. A linear model was selected a priori to characterise the load-velocity relationship, consistent with common practice in resistance exercise research, and no higher-order terms were included due to the absence of an a priori hypothesis of non-linearity. In these models, load (% of 1RM) was included as a fixed effect, while participant was modeled as a random intercept to account for repeated measures. The fastest repetition from each set on all testing occasions was used to develop the load-velocity profiles. Model performance was evaluated using conditional R² values, which accounts for fixed and random effects, and was calculated with the *r.squaredGLMM* function from the *MuMIn* package. Marginal R² values were also calculated to quantify the proportion of variance explained by the fixed effect alone (i.e., %1RM). Sex was not included as a fixed effect in the models due to the small and unbalanced samples, as inclusion of additional between-subject factors under these conditions may result in unstable parameter estimates and model overfitting. Therefore, data were pooled to enable more robust estimation of the within-subject load-velocity relationship.

For each exercise, the group mean and standard deviation of peak velocity were calculated for each load to provide an overview of velocity dispersion across the different relative loads. To improve the validity of the data, outliers were identified and removed through residual analysis, with outliers identified when residuals greater than three standard deviations were found, and visual inspection. Pairwise comparisons between different loads were performed using post hoc tests derived from the linear mixed-effects models with the *emmeans* package. These comparisons accounted for the repeated measures structure and controlled for type I error with Tukey's adjustment. Effect sizes were calculated using Hedge's *g* for each pair of loads, which were included in the analysis to assess the magnitude of differences between loads. Effect sizes were interpreted using thresholds of 0.2–0.59, 0.6–1.19, 1.2–1.99, and >2.0 to signify small, moderate, large, and very large effects, respectively [30]. Predicted velocities and their associated 95% confidence intervals were estimated for specific relative loads at 25%, 45%, 65%, and 80% of 1RM using the *predict* function. Finally, model predictions, confidence intervals, pairwise comparison results, and load statistics (mean and standard deviation of peak velocity) were exported to Excel using the *writexl* package for further analysis.

## Results

The mean and SD 1RM values for each exercise were as follows: clean pull 124 ± 23 kg, hang clean pull 114 ± 22 kg, hang power clean 91 ± 17 kg, hang power snatch 70 ± 15 kg, hang snatch pull 102 ± 19 kg, power snatch 72 ± 16 kg, and snatch pull 106 ± 21 kg. The load-velocity profiles of the seven weightlifting derivatives demonstrated a progressive decline in velocity as the load increased, with *moderate* to *very large* effect sizes across all comparisons. These values can be found in Tables 1 and 2. The conditional R^2^ value was: 0.73 for the clean pull, 0.75 for the hang clean pull, 0.86 for the hang power clean, 0.86 for the hang power snatch, 0.81 for the hang snatch pull, 0.87 for the power snatch, and 0.80 for the snatch pull. The marginal R² values, reflecting variance explained by load (%1RM) alone, were 0.41 for the clean pull, 0.43 for the hang clean pull, 0.44 for the hang power clean, 0.44 for the hang power snatch, 0.26 for the hang snatch pull, 0.51 for the power snatch, and 0.35 for the snatch pull.

**Table 1:** Mean ± SD peak concentric velocities of athletes across 20−90% of one repetition maximum for all exercises. All values are presented as m ∙ s^-1^.

	Exercise						
Load (%1RM)	Power Snatch	Snatch Pull	Hang Power Snatch	Hang Snatch Pull	Hang Clean Pull	Hang Power Clean	Clean Pull
20%	2.94 ± 0.28	2.21 ± 0.49	2.90 ± 0.34	2.24 ± 0.46	2.05 ± 0.45	2.35 ± 0.22	2.03 ± 0.47
40%	2.69 ± 0.21	2.04 ± 0.33	2.72 ± 0.30	2.09 ± 0.33	1.88 ± 0.24	2.20 ± 0.20	1.84 ± 0.28
60%	2.43 ± 0.15	1.83 ± 0.22	2.45 ± 0.19	1.86 ± 0.22	1.65 ± 0.18	2.03 ± 0.16	1.60 ± 0.13
80%	2.21 ± 0.10	1.56 ± 0.16	2.25 ± 0.12	1.60 ± 0.15	1.43 ± 0.14	1.80 ± 0.12	1.35 ± 0.09
90%	2.12 ± 0.10	1.47 ± 0.13	2.15 ± 0.09	1.50 ± 0.16	1.33 ± 0.14	1.69 ± 0.14	1.23 ± 0.09

**Table 2:** P values and Hedge’s *g ±* 95%CL effect size comparisons across all relative loads for all exercises.

	Exercise						
Load Comparisons (%1RM)	Power Snatch	Snatch Pull	Hang Power Snatch	Hang Snatch Pull	Hang Clean Pull	Hang Power Clean	Clean Pull
20 v 40%	<0.001; 1.0 ± 0.58	0.021; 0.41 ± 0.56	<0.001; 0.54 ± 0.58	0.069; 0.37 ± 0.57	0.035; 0.48 ± 0.57	<0.001; 0.7 ± 0.56	0.035; 0.49 ± 0.57
20 v 60%	<0.001; 2.26 ± 0.7	<0.001; 0.99 ± 0.59	<0.001; 1.61 ± 0.65	<0.001; 1.06 ± 0.6	<0.001; 1.15 ± 0.61	<0.001; 1.69 ± 0.63	<0.001; 1.22 ± 0.62
20 v 80%	<0.001; 3.39 ± 0.85	<0.001; 1.76 ± 0.65	<0.001; 2.48 ± 0.75	<0.001; 1.86 ± 0.68	<0.001; 1.85 ± 0.68	<0.001; 3.08 ± 0.8	<0.001; 2.0 ± 0.69
20 v 90%	<0.001; 3.82 ± 0.91	<0.001; 2.04 ± 0.68	<0.001; 2.9 ± 0.82	<0.001; 2.16 ± 0.71	<0.001; 2.13 ± 0.71	<0.001; 3.55 ± 0.88	<0.001; 2.36 ± 0.74
40 v 60%	<0.001; 1.4 ± 0.61	0.003; 0.71 ± 0.57	<0.001; 1.06 ± 0.6	<0.001; 0.82 ± 0.59	0.002; 1.05 ± 0.6	<0.001; 0.99 ± 0.58	0.002; 1.04 ± 0.6
40 v 80%	<0.001; 2.78 ± 0.76	<0.001; 1.78 ± 0.66	<0.001; 1.99 ± 0.69	<0.001; 1.87 ± 0.68	<0.001; 2.27 ± 0.73	<0.001; 2.45 ± 0.72	<0.001; 2.3 ± 0.73
40 v 90%	<0.001; 3.32 ± 0.84	<0.001; 2.2 ± 0.7	<0.001; 2.46 ± 0.76	<0.001; 2.27 ± 0.73	<0.001; 2.75 ± 0.79	<0.001; 2.98 ± 0.8	<0.001; 2.88 ± 0.81
60 v 80%	<0.001; 1.67 ± 0.63	<0.001; 1.39 ± 0.62	<0.001; 1.21 ± 0.62	<0.001; 1.34 ± 0.63	0.002; 1.37 ± 0.63	<0.001; 1.61 ± 0.63	0.002; 2.2 ± 0.72
60 v 90%	<0.001; 2.38 ± 0.71	<0.001; 1.98 ± 0.68	<0.001; 1.95 ± 0.69	<0.001; 1.88 ± 0.68	<0.001; 1.96 ± 0.69	<0.001; 2.26 ± 0.7	<0.001; 3.29 ± 0.87
80 v 90%	0.213; 0.86 ± 0.57	1.0; 0.62 ± 0.57	0.109; 0.94 ± 0.6	0.693; 0.66 ± 0.58	1.0; 0.71 ± 0.58	0.009; 0.83 ± 0.57	1.0; 1.3 ± 0.62

**Fig 1:**
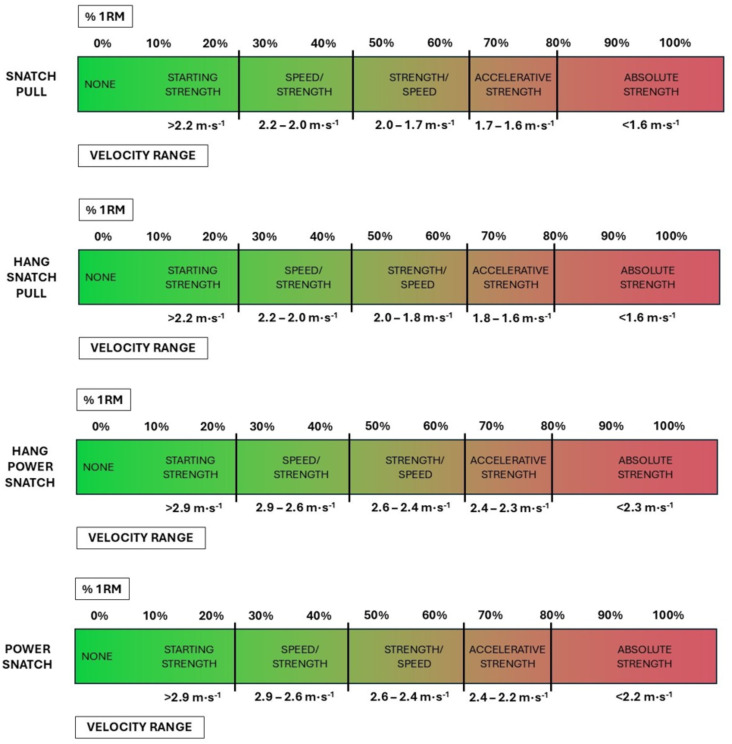
Recommended terminology for peak velocity ranges, relative to percentage of one repetition maximum, for snatch derivative exercises. Note that due to inter-athlete variability these are approximate reference ranges rather than fixed thresholds.

**Fig 2:**
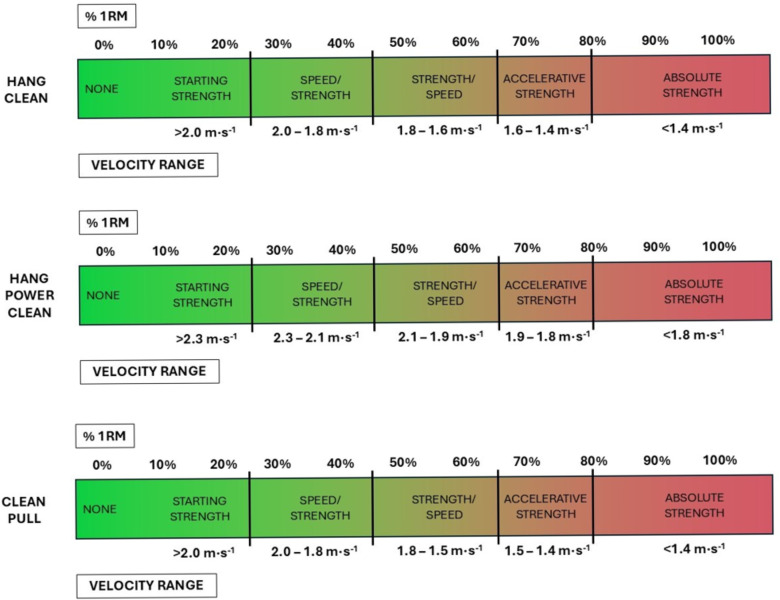
Recommended terminology for peak velocity ranges, relative to percentage of one repetition maximum, for clean derivative exercises. Note that due to inter-athlete variability these are approximate reference ranges rather than fixed thresholds.

Table 3 provides the estimate and confidence limits for the velocities at 25, 45, 65, and 80% of 1RM for each exercise. These velocity estimates are used to inform the velocity zones within Fig 1 and 2, which have been rounded to one decimal place to support practitioner interpretation. The reader should note that these are approximate reference ranges and that inter- athlete variability exists.

**Table 3:** Estimate and 95% confidence limits (CL) for peak velocities from each weightlifting derivative exercise.

	Load (%1RM)			
Exercise	25% Est ± 95%CL (m·s^-1^)	45% Est ± 95%CL (m·s^-1^)	65% Est ± 95%CL (m·s^-1^)	80% Est ± 95%CL (m·s^-1^)
Hang Clean Pull	2.02 ± 0.1	1.81 ± 0.09	1.6 ± 0.09	1.44 ± 0.09
Hang Power Clean	2.33 ± 0.07	2.14 ± 0.07	1.94 ± 0.07	1.8 ± 0.07
Clean Pull	1.99 ± 0.09	1.76 ± 0.08	1.53 ± 0.08	1.36 ± 0.08
Power Snatch	2.87 ± 0.08	2.63 ± 0.07	2.4 ± 0.07	2.23 ± 0.07
Snatch Pull	2.19 ± 0.12	1.97 ± 0.12	1.75 ± 0.12	1.58 ± 0.12
Hang Power Snatch	2.85 ± 0.11	2.63 ± 0.1	2.42 ± 0.1	2.25 ± 0.1
Hang Snatch Pull	2.23 ± 0.13	2.00 ± 0.13	1.78 ± 0.12	1.61 ± 0.13

Est: estimate; 95%CL: 95% confidence limits; m·s^-1^: meters per second.

## Discussion

The aims of this study were to 1) develop load-velocity profiles of seven weightlifting derivatives; and 2) create exercise-specific velocity zones that can be used to guide training prescription. While progressive decreases in velocity occur with load, these changes occur at different rates for each exercise. Furthermore, these profiles demonstrate a range of velocities which are substantially different than previously recommended zones, with all absolute strength velocity zones starting in excess of 1.3 m·s^-1^ (i.e., the previously recommended cut-off for starting strength). This is particularly evident in the power snatch and hang power snatch, with the lightest loads often being more than 3.0 m·s^-1^. It should be noted that previous thresholds are typically based on mean velocity, whereas the present study used peak velocity, which produces systematically higher values, Therefore, these comparisons should be interpreted as conceptual, rather than directly equivalent. These findings provide strong evidence that if generic terms are used for the development of strength qualities (e.g., speed-strength), exercise-specific profiles and velocity zones should be developed. Strength and conditioning practitioners can use the information provided in Fig 1 and 2 to support their training prescription.

The load-velocity profiles for each exercise demonstrate progressive reductions in velocity with increasing load, as well as substantial differences across each exercise. For example, the exercises that included an overhead catch (e.g., the hang power snatch and power snatch), start their absolute strength zone at ~2.2–2.3 m·s^-1^ while the hang pull starts the absolute strength zone at ~1.4 m·s^-1^. These differences between exercises can be attributed to the vastly different technical requirements, ranges of motion, and thus the absolute loads used in these derivatives [31]. For instance, a successful power snatch requires an individual to take the bar from the ground to overhead, while the clean pull requires the bar to travel to approximately waist height. These differences in displacement then require considerably different velocity and momentum for successful execution of the lifts and demonstrates that generic velocity recommendations across all exercises are illogical and often impossible for an athlete to execute.

The velocity zones that are provided in Fig 1 and 2 provide clear practical recommendations for practitioners. The extreme differences between previous guidelines (e.g., absolute strength occurs <0.50 m·s^-1^) and the findings within the current investigation are likely due to the previous recommendations being more aligned with non-ballistic, compound exercises (e.g., the back squat). Nevertheless, non-ballistic compound exercises such as the prone row have velocities at 1RM of ~0.50 m·s^-1^, which indicate the limited application of previous recommendations [17]. It should also be noted that the velocity variable (i.e., mean vs peak) used to quantify performance needs to be considered, with peak velocity advised for weightlifting derivatives due to their ballistic intent [1,16]. By acknowledging the specificity of exercise velocity ranges and variables, improved recommendations for the development of physical traits can be made. However, as the present study is descriptive, the extent to which training within these velocity zones elicits specific strength or performance adaptations remains unclear and warrants investigation in longitudinal intervention studies.

While the findings in this manuscript provide clear evidence supporting an exercise-specific approach to monitoring training with velocity, several limitations should be acknowledged. First, peak velocity is inherently phase-specific and sensitive to subtle changes in movement strategy and technical execution, particularly in weightlifting derivative exercises where alterations in bar path, timing of joint extension, or intention to accelerate may substantially influence the recorded outcome. Although peak velocity has practical appeal for practitioners and is the recommended velocity variable when using these types of exercise, it should be interpreted as a proxy of performance rather than a comprehensive descriptor of the full concentric movement. Second, velocity was sampled at 30 Hz, which may underestimate true peak values, especially in ballistic movements where velocity changes rapidly over short time frames. Third, the decision to retain the fastest repetition at each load reflects common applied practice and aligns with the conceptual use of velocity-based training to capture maximal intentional performance. However, this approach may amplify the influence of trial-to-trial technical variability and does not account for within-set or within-load consistency, which may be relevant for practitioners interested in fatigue- or reliability-related outcomes. Fourth, the velocity zones and associated terminology in this manuscript provide clear guidelines, yet they are just guidelines. Velocity is a continuous variable and having clear discrete zones that all athletes work in is ill-advised. The confidence limits provided in Table 3 help to demonstrate the uncertainty around these general zones and the R² values emphasise that additional factors (e.g., muscle typology, sex, or motivation) may also influence the load–velocity profile of an athlete. Furthermore, the cohort used within this study were highly trained. Therefore, it should be acknowledged that lesser trained individuals may have different velocity zones. Fifth, some of the movements examined in this study (e.g., the clean pull and snatch pull) are traditionally prescribed as a relative percentage of the full clean or snatch. In contrast, this study progressively loaded each exercise until the maximum load that could be performed with proficient technique was identified (i.e., the movement-specific 1RM). All repetitions were visually inspected by an accredited strength and conditioning practitioner to ensure technical proficiency, allowing full load–velocity profiles to be established for each exercise. Nevertheless, it is acknowledged that this approach differs from how some practitioners prescribe these movements in applied settings and may influence the external validity of the resulting velocity zones. Sixth, the load–velocity profiles presented in this study represent a cross-sectional snapshot of performance. It is plausible that targeted training interventions (e.g., strength focused training) may alter the velocity associated with a given %1RM over time through changes in neuromuscular function or technical execution. Finally, while previous research has investigated the validity and reliability of the Perch for monitoring resistance training, it is plausible that there are systematic differences across different devices. Therefore, if these velocity zones are used, future research should investigate whether these zones are device specific.

In conclusion, this investigation provides the load-velocity profile of seven weightlifting derivatives and provides velocity ranges that can support coaches in targeting strength related physical qualities. The findings demonstrate that due to the vastly different load-velocity profiles of each exercise, previously recommended zones are impractical, and in many cases are likely impossible to attain. Consequently, it is advised that generic strength terms (e.g., accelerative strength, speed-strength) are more closely aligned with the real-world load-velocity profiles that athletes exhibit. Additionally, practitioners can use the findings from this investigation to help target specific strength qualities. However, it is important to note that load-velocity profiles do differ across athletes, and that the ranges provided are merely guidelines that can support practice.

### Practical applications

The current findings provide the load-velocity profiles of seven commonly used weightlifting derivatives. Furthermore, this study provides exercise specific velocity zones that align with terms such as starting strength, speed-strength, strength-speed, accelerative strength, and absolute strength. Fig 1 and 2 provide a practical reference for these ranges across each exercise, which practitioners may use to inform load prescription and monitoring within velocity-based training frameworks. However, these values should be interpreted as general guidelines rather than exact targets, as there can be between-athlete variability. As such, individual calibration is recommended to ensure alignment with each athlete’s unique load-velocity profile.. For example, a practitioner may want to target accelerative strength with an athlete during the hang clean, thus may want to target loads that allow a peak velocity of 1.4–1.6 m·s^-1^. However, these ranges should be adjusted based on the individual athlete, and practitioners are encouraged to use them as a starting point rather than prescriptive thresholds.

## Supporting information

S1 File Inclusivity-in-global-research-questionnaire. (DOCX)
